# Using EEG to Predict Clinical Response to Electroconvulsive Therapy in Patients With Major Depression: A Comprehensive Review

**DOI:** 10.3389/fpsyt.2021.643710

**Published:** 2021-06-24

**Authors:** Louis Simon, Martin Blay, Filipe Galvao, Jerome Brunelin

**Affiliations:** ^1^Centre Hospitalier Le Vinatier, Bron, France; ^2^INSERM, U1028, CNRS, UMR5292, Lyon Neuroscience Research Center, PSYR2 Team, Lyon, France; ^3^Lyon University, Université Lyon 1, Villeurbanne, France

**Keywords:** depression, biomarker, major depression, prediction, EEG, ECT

## Abstract

**Introduction:** An important approach to improve the therapeutic effect of electroconvulsive therapy (ECT) may be to early characterize patients who are more likely to respond. Our objective was to explore whether baseline electroencephalography (EEG) settings before the beginning of ECT treatment can predict future clinical response to ECT in patients with depressive disorder.

**Methods:** We conducted a systematic search in the MEDLINE, EMBASE, PsycINFO, Web of Science, and Cochrane Central Register of Controlled Trials (CENTRAL) databases to identify studies using EEG in adults with depressive disorder treated by ECT. To investigate the predictive value of baseline EEG on clinical outcomes of ECT, we extracted from the retrieved studies and qualitatively described the association between the baseline EEG markers characteristics and the rates of future responders and/or remitters to ECT.

**Results:** The primary search yielded 2,531 potentially relevant citations, and 12 articles were selected according to inclusion criteria. Most of the studies were prospective studies with small sample size. Sociodemographic and clinical characteristics of patients, ECT settings, EEG settings, and outcomes were heterogeneous. Event-related potential (ERP) paradigms were used in three studies, polysomnography was used in three studies, and the six other studies used EEG to measure cerebral connectivity and activity.

**Conclusions:** P300 amplitude, coherence, and connectivity measures were correlated with remission in patients with depression treated by ECT. Sleep EEG recordings seemed not to be correlated with remission after ECT. Further prospective studies with large sample size are needed to determine optimal EEG parameters associated with clinical response to ECT in depressive disorder.

**Systematic Review Registration:** PROSPERO CRD42020181978.

## Introduction

Electroconvulsive therapy (ECT) is among the oldest and most effective therapy for the treatment of depressive disorders, more effective than pharmacotherapy (1). ECT has demonstrated response rates between 60 and 80% and remission rates between 50 and 60% (2). Indications in depressive disorders are life-threatening psychotic, suicidal features and treatment-resistant depression. However, disabling cognitive side effects limit the use of ECT in clinical settings. One important approach to improve the therapeutic effect of ECT as well as to reduce the impact of side effects and the stigma associated with ECT may be to increase personalization of the treatment. The identification of patients sharing common baseline characteristics that should predict remission after ECT treatment seems to be a suitable approach. A recent meta-analysis identified that the presence of psychotic features, older age, and higher severity of depression were associated with ECT response (3). Pinna and colleagues also reported that older age, presence of psychotic and melancholic depression, a high severity of suicide behavior, and speed of response are associated with clinical response to ECT in depression (4). Regarding the influence of age, Heijnen and colleagues found that the association between age and ECT efficacy was significantly mediated by psychomotor retardation and a lesser extent by psychotic features (5). Pinna and colleagues find a link between response to ECT and the presence of pretreatment hyperconnectivity between brain regions involved in the pathophysiology of depression and reduced glutamine/glutamate levels, especially in the anterior cingulated cortex (ACC), measured with both magnetic resonance or nuclear imaging modalities (4). However, the usefulness at an individual level and the cost and accessibility of such biological predictors limit their use at a large scale in clinical settings.

Electroencephalography (EEG) is a widely available and low-cost imaging technique. In patients with depressive disorders, sleep EEG, resting-state EEG, quantitative EEG (QEEG), connectivity measures, and event-related potentials (ERPs) have been used to discriminate characteristics of patients from those of healthy subjects and also to monitor and predict the clinical changes and outcome of various antidepressive treatments in patients (6–8). EEG can be performed before ECT treatment (i.e., baseline), during ECT (i.e., ictal), between ECT sessions (i.e., interictally), and after ECT treatment (9). In 2006, a systematic review of the literature on ictal EEG characteristics during ECT identified postictal suppression, postictal coherence and amplitude, amount of slowing and time to onset of slowing, global EEG power, largest Lyapunov exponent, and Strength Symmetry index as predictors of ECT response (10). In 2020, Janoushek and colleagues retrospectively evaluated an algorithm with seizure quality features to predict clinical response (11). Some researchers focused on EEG changes between before and after ECT treatment. As an example, it has been reported that ictal interhemispheric coherence in the theta and alpha frequency bands can increase over the course (12). Nevertheless, one concern that preoccupies ECT researchers is whether any EEG changes can predict treatment response or are simply an epiphenomenon (9).

The objective of this review was therefore to explore whether EEG undertaken before the beginning of ECT treatment can be used to predict ECT clinical response in patients with depressive disorder. To increase our relevance, we focused only on baseline EEG, as did studies searching clinical, serological, or functional MRI (fMRI) predictors.

A systematic search in the literature was conducted to identify studies recording baseline EEG in adults with depressive disorder who received ECT in order to establish the predictive value of baseline EEG on clinical outcome in this indication.

## Methods

This study was performed according to the Preferred Reporting Items for Systematic Reviews and Meta-Analyses guidelines (13). Details of the protocol were registered on PROSPERO and can be accessed at www.crd.york.ac.uk/PROSPERO/display/record.php?ID=CRD42020181978

### Eligibility Criteria

Inclusion criteria were the following: (a) Articles should be published in peer-reviewed journals in the English language. (b) Randomized controlled trial, cohort study, case–control study, and cross-sectional study were included. Reviews, case reports and series, qualitative studies, and meeting abstract without peer reviewing were excluded. (c) Articles should include original baseline EEG measures recorded before any ECT, either sleep EEG, ERP, or resting-state EEG. Studies with only ictal EEG (during ECT) or differences between post- and pre-EEG (i.e., focusing on EEG changes during ECT course) were not included. (d) Studies should include adults with unipolar or bipolar depressive disorder meeting recognized diagnostic criteria who were treated by ECT. (e) Studies should provide comprehensive and detailed findings.

### Search Strategy

The search was conducted on four bibliographic databases: MEDLINE, EMBASE, PsycINFO, Web of Science, and one trial register, Cochrane Central Register of Controlled Trials (CENTRAL), from the date of their inception to May 7, 2020. Reviews and bibliographies were scrutinized for further relevant studies. Unpublished studies were sought with CENTRAL.

The search terms included “Electroconvulsive therapy” and “Electroencephalogra^*^” and was constructed with free text, abbreviations (“EEG” and “ECT”), and expanded medical subject headings. A copy of the search strategy can be accessed online on PROSPERO.

Two investigators (MB and LS) independently screened the results according to the eligibility criteria and selected studies for inclusion with primary (title and abstract) and secondary (full text) screening before comparing results. They have used rayyan, a web app for systematic reviews (14). Each reviewer was blinded to each other's decisions. Disagreements were resolved by discussion with a third investigator (FG).

### Data Extraction and Quality Assessment

Data were extracted using a predefined and pilot-tested data extraction form, including quantitative results. The online extraction tool, Systematic Review Data Repository Plus, was used. Main outcomes were the changes in depressive symptoms after ECT treatment (defined clinically by the physician or with standardized psychometric scales) and spectral analysis of EEGs (quantitative EEG), EEG source localization, connectivity measures, EEG-evoked potentials, sleep-related EEG measures, and any combinations of quantitative and qualitative EEG features or EEG-based machine-learning approaches. Information on study designs, ECT settings, ECT-associated side effects, patient clinical characteristics, and treatment were also extracted to assess heterogeneity and confounding factors.

Studies were reviewed for quality and bias risk using the Standard Quality Assessment Criteria for Evaluating Primary Research Papers (the QualSyst tool) (15).

### Data Analysis

We qualitatively described results from selected studies because quantitative information did not allow us to perform a meta-analysis. Data were synthesized using three outcome categories: baseline EEG-evoked potentials and associated measures [i.e., transcranial magnetic stimulation-EEG (TMS-EEG)], baseline sleep-related EEG measures, and other baseline EEG-derived measures.

## Results

### Study Selection

**Figure 1:**
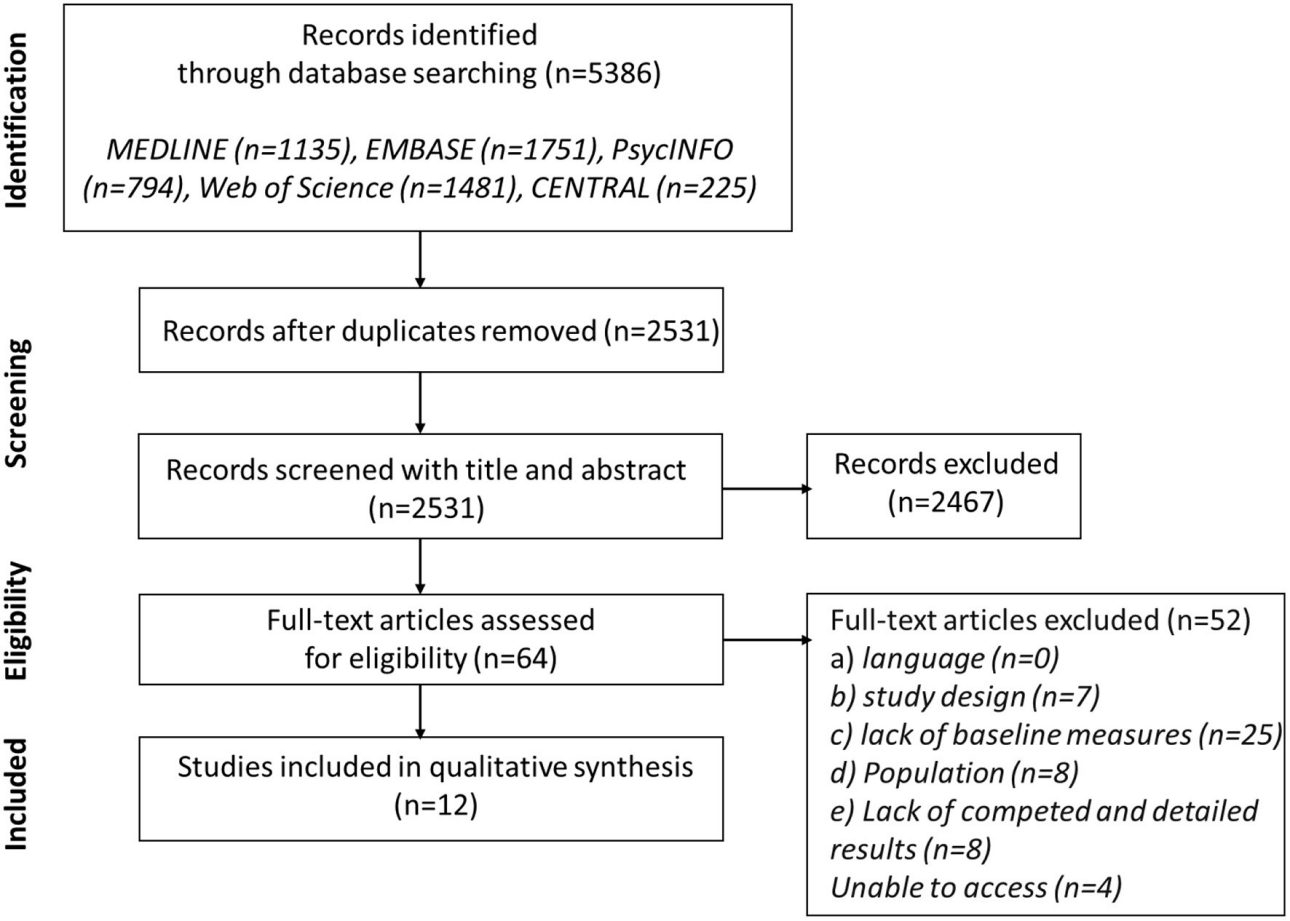
Preferred reporting items for systematic reviews and meta-analysis (PRISMA) flow diagram of search.

The primary search yielded 2,531 results. Among them, we excluded 2,467 after reviewing their title/abstract and 52 after full-text review according to our eligibility criteria. Twenty-five articles were excluded due to lack of baseline EEG data and focus on EEG changes during ECT course (differences between post and pre). It was the first reason for exclusion. Four articles were excluded due to inaccessibility; they were all written before 1995 (1994, 1980, 1972, and 1970). Twelve articles written between 1989 and 2020 were selected for inclusion. Figure 1 shows the Preferred Reporting Items for Systematic Reviews and Meta-analysis (PRISMA) flow chart detailing information through the different phases.

### Study Characteristics

Key characteristics of the included studies are presented in Table 1. Nine studies were prospective, with a control group in two cases but not used in data analysis. Three studies were retrospective. Generally, the sample size from the selected studies were quite small, with a mean number of patients varying from 10 (18, 26) to 100 (23) (mean = 30.1). Sociodemographic characteristics of patients were heterogeneous across studies with mean age varied from 40.7 (16) to 75.5 (25) [mean = 54.9, not available (N/A) = 3 studies] and mean percentage of male patients varied from 26.7 (21) to 70.0% (27) (mean = 44.7%, N/A = 4).

**Table 1:** Study characteristics, results, and risk of bias within studies.

**Reference**	**N**	**Age years**	**M%**	**Diagnoses**	**ECT settings**	**EEG**	**Comparator**	**Outcomes**	**Results**	**B**
(16)	17	40.7	47.1	DSM-III-R Depressives *N =* 4 Bipolar *N =* 8 recurrent *N =* 5 single episode All melancholia without psychotic symptoms	BFT SW 2 (*N =* 4) 3 (*N =* 13) /w ECT 7.3	P300: Auditory task with oddball paradigm Electrode Cz BF 0.5–35 Hz	Rapid responders (HDRS = 7, 2nd week) vs. slow (HDRS = 7, 3rd and 4th week)	P300 amplitude and latency	Amplitude smaller in slow responders p < 0.02 t = 2.62 Latency NS	17/24
(17)	23	44.0	65.2	DSM-III-R *N =* 17 unipolar psychotic major depressions *N =* 6 psychotic depressive episodes of non-unipolar disorder	BT 3/w ECT 7.7	P300: auditory task with oddball paradigm Fz, C3, Cz, C4, Pz BF 0.3–50 Hz	N/A	Correlation reduction in HDRS AND P300 amplitude (C3, T3)	Spearman C3 r = −0.615, p = 0.036 Spearman T3 NS (r = −0.595, p = 0.054)	16/24
(18)	10	54.5	50.0	DSM 5 MDD	BL BPCC 2–3/w ECT 5.5	TMS-EEG 27 electrodes focus on the vicinity of C3 and Oz BF 0.1–30 Hz	N/A	Correlation post-MADRS AND premaximum alpha PLVs OR maximum alpha PLFs	Pearson's (r) post-MADRS AND PLVs (between motor and visual areas) OR PLFs (for motor AND visual areas): all NS (3) Pre-ECT vs. post-ECT MADRS scores was not significant	9/24
(19)	41	65.2	24.4	RDoC MDD (1978) (20) *N =* 35 endogenous subtype *N =* 16 psychotic subtype	SW and BP *N =* 21 UL *N =* 19 BL *N =* 1 unknown	Polysomnographic recordings Electrodes C3/A2 BF 0.3–30 Hz	Good vs. poor response (HDRS <11 vs. ≥11)	Association between response and sleep architectures and REMS parameters	Two-factor ANOVA sleep architectures (Stage 2%, Delta%) all NS (2) Two-factor ANOVA REMS parameters (latency, number of periods, total, density) NS (4)	16/24
(21)	15	58.8	26.7	ICD-10 Severe major depression *N =* 14 unipolar *N =* 1 bipolar	RUL 2–3/w ECT 7.3	Polysomnographic recordings Electrodes C3-A2 and C4-A1	Remitters vs. nonremitters at 6 months (21-items HDRS score <8 vs. >8)	Association between remission and REM density of first REMS period	ANOVA Time × Group p = 0.04 *post-hoc* t-tests: stable remitters higher REMS density Correlation REMS density, HDRS after ECT r = −0.54; p = 0.049, 6 months after ECT NS t-test patients with ≤ 7% or >7% of REMS density for HDRS pre–post p = 0.02 for pre–post (6 months) and for HDRS and BDI post (6 months) NS (3)	17/24
(22)	16	N/A	N/A	DSM-IV-TR Major depressive episode without psychotic features	No ECT	Polysomnographic recordings 19 electrodes	Potential response to ECT (Newcastle subscale <1 vs. ≥1)	Correlation potential response AND REMS + SWS time/frequency domain	Spearman SWS time domain 0.588 frequency domain β 0.644, σ 0.588, α 0.728, θ 0.728, δ 0.756 (all p <0.05) Spearman REMS time domain and frequency domain (β, σ, α, θ, δ) all NS (6)	12/24
(23)	100	N/A	N/A	Melancholic depression Schizoaffective disorder with depression	N/A	EEG intermittent photic stimulation and hyperventilation 17 electrodes	Retrospective remission with/without confusion, improvement, little or no change	Association between response and EEGs retrospectively classified (normal, limit, abnormal)	Chi-square EEG, clinical response N.S Chi-square EEG (normal and borderline vs. focal and generalized EEG slowing), clinical response EEG slowing related to incomplete remission p = <0.05, X^2^ = 4.05, df 1	9/24
(24)	21	Range 54–86	33.3	DSM III-R *N =* 13 depression, recurrent, severe, without psychotic features *N =* 8 with psychotic features	UL or BL BPCC ECT range 2–12	Eyes-closed EEG interhemispheric coherence between 8 monopolar lead pairs	Good vs incomplete ECT response (+60% HDRS, HDRS <15 vs. −60% HDRS)	Association between response and interhemispheric coherence between homologous leads	rmANOVA interhemispheric coherence significant only for delta band (p = 0.01, *post-hoc* differences between F3–F4, F7–F8, and T3–T4) Correlation frontal interhemispheric coherence in delta band and % HDRS decrease r = 0.46, p <0.05	14/24
(25)	30 38	75.5 54.4	N/A	DSMIII-R Major Depression	Study 1: RUL SP 3/w Study 2: BT BP	Eyes-closed EEG 21 electrodes BF 1–70 Hz and 60 Hz Notch filter	N/A	Regression model of post–pre MADRS and baseline prefrontal and frontocentral delta coherence	Study 1: p for model = 0.01 association R^2^ = 0.44 magnitude M = −6.7 Study 2: p for model = 0.008 association R^2^ = 0.16 magnitude M = −5.44	12/20
(26)	10	59.2	50.0	DSM-IV MDD Bipolar I Disorder	*N =* 8 BL *N =* 2 RUL	Eyes-closed EEG with regional variables 35 electrodes BF 0.3–50 Hz	N/A	Linear regression model of % decrease in HDRS and cordance measure, QEEG absolute and relative power	Central cordance, HDRS r = 0.80, p = 0.005 (still significant after Bonferroni correction) QEEG and cordance (other regions), HDRS NS Other regions: prefrontal, frontocentral, left and right temporal, left and right parietal, occipital	13/24
(27)	17	45.6	70.0	DSM-IV Major depression with psychotic features	BPCC *N =* 16 RUL *N =* 7 to BL *N =* 1 BL ECT 12.4	Eyes-closed EEG (LORETA) 19 electrodes BF 1–70 Hz	+50 vs. −50% improvement in SAPS	Subgenual ACC theta activity before ECT	Lower in better responders p <0.001, t = 6.1 Subgenual ACC theta hypoactivity predicted change in psychotic symptoms rho = 0.594 (p <0.007)	21/24
(28)	53	51.2	35.8	ICD-10 MDD Recurrent MDD Depressive episode in a bipolar disease	ECT 10.3	Eyes-closed EEG (LORETA) 26 electrodes BF 0.5–70 Hz and 60 Hz Notch filter	Responder vs. nonresponder (CGI-E 1 or 2 vs. 3 or 4)	Correlation between response and linear connectivity between nodes for alpha 1 and alpha 2 frequency band	Alpha 2 significant effect with lower connectivity for responder Alpha 1 NS Spearman rank correlation: role of frontal left side associations and frontal interhemispheric correlations in CGI improvement	15/24

*Unless otherwise specified, numeric data are means and standard deviations (N, number of patients; M, male (%); Me, median; NS, not significant; B, risk of bias; N/A, not available)*.

*BF, bandpass filtered; BFT, bifrontotemporal; BP, brief pulse; BPCC, brief pulse constant current; BT, bilateral; LORETA, low-resolution electromagnetic tomography; MDD, major depressive disorder; PLF, phase locking factor; PLV, phase locking value; REMS, rapid eye movement sleep; RUL, right unilateral; S, sedatives (including benzodiazepines and anxiolytics); SAS, sleep apnea syndrome; SP, standard pulse; SW, sine wave; SWS, slow wave sleep; UL, unilateral; /w, number of ECT per week*.

Regarding diagnoses, all patients presented with depressive episodes. Eleven studies included unipolar patients (with or without bipolar patients); four included both unipolar and bipolar patients. Episodes were specified as recurrent in three studies and melancholic in two studies. Four studies included depressive patients with psychotic features, and one included patients with schizoaffective disorder with depression. Severity of illness before ECT was quite homogeneous with mean scores at the 17-item Hamilton Depressive Rating Scale (HDRS_17_) ranging from 26.9 (16) to 28.7 (26) (*N* = 3 studies), from 29.3 (17) to 29.7 (21) (*N* = 2) at 21-item HDRS and from 34.0 (25) to 38.5 (17) at the Montgomery–Asberg Depression Rating Scale (MADRS) (*N* = 2, excluding the mean score of 18.3 in Miyauchi and colleagues (18), where pre-ECT vs. post-ECT MADRS scores were not significant. Non-psychiatric comorbidities were described only in one study (21). Regarding associated medication, patients received psychotropic drugs in seven studies and did not received drugs in three studies (N/A = 2): antidepressants in five studies, antipsychotics in five studies, benzodiazepines and anxiolytics in five studies, and mood stabilizers in four studies.

Electrode placement was bitemporal in three studies, right unilateral in one study, and bitemporal or right unilateral in five studies (N/A = 2). ECT frequency was three times a week in one study and two or three times a week in three studies (N/A = 7). One study used as comparator the Newcastle subscale to predict potential response to ECT, and ECT treatment was not described (22).

Event-related potential (ERP) paradigms EEG experiments were used in three studies to find a biomarker associated with ECT response. Two focus on the auditory P300 ERP (16, 17) and one on the combination of TMS with EEG (18). Three studies used EEG among other polysomnographic recordings (19, 21, 22). The oldest study, published in 1989, used EEG intermittent photic stimulation and hyperventilation (23), and the five other studies used eyes-closed resting-state EEG (24–28). Two eyes-closed EEG studies computed three-dimensional space with low-resolution electromagnetic tomography (LORETA) to analyze data. Electrodes numbers and bandpass filters varied according to regions and frequency bands of interest.

### Risk of Bias Within Studies

The QualSyst tool scores reflected key components of internal study validity (15). Scores varied from 0.38 (18, 23) to 0.88 (27) (mean = 0.60). On average, in the 12 studies, the limited size of the sample and the lack of control for confounding were the most frequent cause of the decrease of scores. Patient characteristics description, blinding of investigators, and the lack of sufficient details in results (e.g., estimate of variance) were others internal limits.

### Synthesis of Results

In auditory ERP studies, authors found a smaller P300 amplitude over Cz in rapid responders to ECT (HDRS = 7, second week) compared to slow responders (HDRS = 7, third and fourth week) and a correlation between the P300 amplitude and the reduction of depressive symptoms after ECT (16, 17); the smaller the P300 over C3, the greater the reduction in HDRS scores. Results from the study using TMS-EEG to measure baseline characteristics did not claim for the interest of this measure as a predictive marker of response to ECT (18).

In polysomnographic studies, results on rapid eye movement sleep (REMS) were contradictory. Hein and Grunhaus did not find any significant association between response or potential response to ECT and REMS parameters (including density) or REMS time and frequency domain (19, 22). Göder and colleagues found an association between remission and REM density, but this result was not consistent in the study, and multiple testing was not considered (21). Hein and colleagues found correlations between potential response to ECT (defined using the Newcastle subscale) and slow wave sleep (SWS) time and frequency domain (22). This result was not found with real response to ECT. Results on sleep architectures (Stage 2%, Delta%) were not significant (19).

Drake and colleagues did not find significant association between clinical response to ECT and retrospectively classified EEG with intermittent photic stimulation and hyperventilation. Focal and generalized EEG slowing was related to incomplete remission but not to ECT resistance (little or no change) (23).

Two eyes-closed EEG studies found an association between clinical response and baseline delta coherence: interhemispheric between F3–F4, F7–F8, and T3–T4 (24) or prefrontal and frontocentral (25). Regarding alpha 2 frequency band, Kirsten and colleagues found a lower linear connectivity in responders and a role of frontal left side associations and frontal interhemispheric correlations in clinical improvement. Results on alpha 1 frequency band were not significant (28). Stubbeman and colleagues found a significant association between the decrease in HDRS and central cordance; other regions and QEEG measures were not significant (26). With LORETA analyses, McCormick and colleagues found lower subgenual ACC theta activity in better responders to antipsychotic ECT effect (27).

## Discussion

The current literature review aimed to identify whether baseline EEG before the beginning of ECT would be useful to predict ECT clinical response in adults with depressive disorder. Outcomes of baseline EEG measures taken into account in this review were sleep-related EEG measures, EEG-evoked potentials, TMS-EEG, and other baseline EEG-derived measures. Studies were mainly prospective with small sample sizes. Sociodemographic and clinical characteristics of patients, ECT settings, EEG settings, and outcomes were heterogeneous. It is a limitation to data synthesis, and it increases risk of results linked to confounding factors across studies (e.g., EEG results with patients suffering from depression with psychotic features could be driven by psychotic features and not by ECT response). Another limitation is the use of psychotropic drugs in seven studies. In a recent systematic review evaluating the impact of psychotropic drugs, alpha, beta, delta, and theta waves were impact independently and differently from each other (29). Drugs could distort baseline features and mask a moderate predictive effect of EEG due to difference in prescription across studies. The difference in diagnoses across studies are a source of heterogeneity, too. Nevertheless, ECT settings are similar in depressive patients with unipolar and bipolar disorder or with psychotic features. Moreover, in a recent meta-analysis, Bahji and colleagues found equivalent remission rates in depressive patients with bipolar or unipolar disorder (30). Due to the ECT treatment and response similarities for depressive patients, we found it useful to include them all in our review.

The P300 wave is a parietocentral positive deflection in EEG occurring when an informative-relevant task stimulus is detected (31). Various components of the P300 waveform were associated with different neuropsychological processes and particularly attentional processes (32). These markers are not expected to be pathology specific and show similarities between depressive disorder, bipolar disorder, and schizophrenia (32, 33). Smaller baseline P300 amplitudes were significantly decreased only in patients with psychotic features (34) and has been found in antidepressant treatment non-responders compared to responders (35). In our review, results seemed contradictory with smaller P300 amplitudes correlated to slower (16) or best (17). ECT responses for auditory tasks with oddball paradigm. These results could be linked to differences in the recording sites: Fz, Cz, Pz, C3/4, and P3/4 for response to antidepressant treatment (35) and only at Cz or C3 for response to ECT (16, 17). These results highlight the need to have more reproducible EEG and ERP settings with homogeneous patients.

Depression is associated with a decrease in SWS production and disturbed REMS regulation (36). Shortened REM latency (i.e., the interval between sleep onset and the occurrence of the first REM period) increased REMS duration and increased REM density (i.e., the frequency of rapid eye movements per REM period) might predict relapse and recurrence (36). In 2017, in a meta-analysis on biomarkers of response to pharmacological antidepressant treatment, polysomnographic sleep measures were reported as one of the two best objective markers of clinical response (37). In another meta-analysis investigating abnormalities of sleep-EEG measures in patients with depression, it was reported that all the baseline EEG abnormalities were normalized after antidepressive treatment in remitters except REM density and SWS (38). Despite these promising results, in our systematic review with regards to ECT response, results on SWS sleep and REMS (density, time, and frequency domains measured with EEG) were contradictory, and data synthesis was not possible. Other studies with larger sample size and reproducible settings are needed.

Coherence function measures the degree to which EEG signals recorded simultaneously are similar in their patterns of amplitude fluctuation: it measures the correlation between two signals as a function of the frequency components that they contain (39). Delta oscillations are involved in motivational processes attention and salience detection (40). Scangos and colleagues extended first findings of interhemispheric coherence in the delta frequency band to that of intrahemispheric coherence and to prefrontal sites (25). With LORETA analysis, Kirsten and colleagues found another association between a connectivity measure, as coherence, and clinical response to ECT (28). These results highlight that direct and indirect measure of connectivity might be a promising way to find a marker of ECT response.

Cordance is a QEEG measure that combines the amount of power in an EEG frequency band at a given electrode (EEG absolute power) and percentage of power contained in a frequency band in relation to the total power (EEG relative power) (41). Cordance indicates the nature of electrical activity to characterize underlying cortical metabolism and perfusion (42). Stubbeman and colleagues found an association between clinical response and central cordance (26). Most recently, in a magnetoencephalography study in late-life depression, no difference was found at baseline between early responders and non-responders in mean frontal theta cordance (43). These results are contradictory. Nevertheless, cordance reflects the pathophysiology of depression with unbalanced activity and might be another promising marker of ECT response.

McCormick and colleagues found lower subgenual ACC theta activity in better responders (27). Contrary to the McCormick results with ECT, Pizzagalli and colleagues showed higher theta activity in the rostral anterior cingulate in patients with better response to antidepressant treatment (44). Moreover, in a recent systematic review with four studies evaluating rostral theta activity in ACC as biomarker of antidepressant pharmacological treatment response, an increase in theta (slow) waves was found to be predictive (37). Further studies are needed to explore the association between ACC theta activity and ECT response even if this region might be associated with the response to depression.

The main limitation of the current review is the heterogeneity across studies that increase the risk of confounding factors. There is also a high risk of publication bias, limiting the generalization of our results. We retrieved studies investigating both sleep-related EEG, EEG-evoked potentials, and EEG with measures reflecting connectivity and activity unbalance in depression. By focusing on strict EEG baseline data, we drastically limited other confounding factors and risk to consider epiphenomenon, not associated with direct ECT response. The most promising outcomes were P300 amplitude in cortical regions (C3, Cz) measured during auditory task with oddball paradigm, delta coherence in prefrontal and frontocentral regions or between hemispheres measured with eyes-closed EEG, and connectivity in widespread cortical areas within the alpha 2 band analyzed with LORETA. Results on theta activity in the ACC are contradictory with those observed in patients with depression. Sleep EEG recordings seem not to predict remission after ECT. Other prospective studies with larger sample size and more reproducible design are needed. Clinical characteristics of depressed patients (i.e., unipolar or bipolar, single or recurrent episodes, with psychotic or melancholic features, baseline illness severity and comorbidities, psychotropic drugs) and ECT settings (i.e., unilateral or bilateral, frequency, number of sessions) must be considered to not measure an effect driven by a confounding factor. Moreover, more similarities in EEG settings and outcomes across studies would be necessary to have a potential marker available in current clinical practice.

## Data Availability Statement

The original contributions presented in the study are included in the article/supplementary material, further inquiries can be directed to the corresponding author/s.

## Author Contributions

LS: conceptualization, investigation, methodology, data curation, writing—original draft, and visualization. MB: investigation and data curation. FG: conceptualization, validation, writing—review editing, and supervision. JB: conceptualization, methodology, writing—review editing, and supervision. All authors contributed to the article and approved the submitted version.

## Conflict of Interest

The authors declare that the research was conducted in the absence of any commercial or financial relationships that could be construed as a potential conflict of interest.

## References

[1] UK ECT Review Group. Efficacy and safety of electroconvulsive therapy in depressive disorders: a systematic review and meta-analysis. Lancet. (2003) 361:799–808. 10.1016/S0140-6736(03)12705-5 12642045

[2] Weiner RD Reti IM. Key updates in the clinical application of electroconvulsive therapy. Int Rev Psychiatry. (2017) 29:54–62. 10.1080/09540261.2017.1309362 28406327

[3] van Diermen L van den Ameele S Kamperman AM Sabbe BCG Vermeulen T Schrijvers D. Prediction of electroconvulsive therapy response and remission in major depression: meta-analysis. Br J Psychiatry. (2018) 212:71–80. 10.1192/bjp.2017.28 29436330

[4] Pinna M Manchia M Oppo R Scano F Pillai G Loche AP. Clinical and biological predictors of response to electroconvulsive therapy (ECT): a review. Neurosci Lett. (2018) 669:32–42. 10.1016/j.neulet.2016.10.047 27793702

[5] Heijnen WTCJ Kamperman AM Tjokrodipo LD Hoogendijk WJG van den Broek WW Birkenhager TK. Influence of age on ECT efficacy in depression and the mediating role of psychomotor retardation and psychotic features. J Psychiatr Res. (2019) 109:41–7. 10.1016/j.jpsychires.2018.11.014 30472527

[6] Mumtaz W Malik AS Yasin MAM Xia L. Review on EEG and ERP predictive biomarkers for major depressive disorder. Biomed Signal Process Control. (2015) 22:85–98. 10.1016/j.bspc.2015.07.003

[7] Olbrich S van Dinteren R Arns M. Personalized medicine: review and perspectives of promising baseline eeg biomarkers in major depressive disorder and attention deficit hyperactivity disorder. Neuropsychobiology. (2015) 72:229–40. 10.1159/000437435 26901357

[8] Olbrich S Arns M. EEG biomarkers in major depressive disorder: discriminative power and prediction of treatment response. Int Rev Psychiatry. (2013) 25:604–18. 10.3109/09540261.2013.816269 24151805

[9] Farzan F Boutros NN Blumberger DM Daskalakis ZJ. What does the electroencephalogram tell us about the mechanisms of action of ECT in major depressive disorders? J ECT. (2014) 30:98–106. 10.1097/YCT.0000000000000144 24810774

[10] Mayur P. Ictal electroencephalographic characteristics during electroconvulsive therapy: a review of determination and clinical relevance. J ECT. (2006) 22:5. 10.1097/01.yct.0000235922.14623.39 16957539

[11] Janouschek H Langbehn DR Nickl-Jockschat T Grözinger M. The impact of seizure quality on ect treatment efficacy. Psychiatry Res. (2020) 293:113466. 10.1016/j.psychres.2020.113466 33198041

[12] ten Doesschate F van Wingen GA de Pont BJHB Arns M van Waarde JA. The longitudinal effects of electroconvulsive therapy on ictal interhemispheric coherence and its associations with treatment outcome: a naturalistic cohort study. Clin EEG Neurosci. (2019) 50:44–50. 10.1177/1550059418781698 29929395

[13] Moher D Liberati A Tetzlaff J Altman DG. Preferred reporting items for systematic reviews and meta-analyses: the PRISMA statement. PLoS Med. 6:e1000097. 10.3736/jcim20090918 19621072 PMC2707599

[14] Elmagarmid A Fedorowicz Z Hammady H Ilyas I Khabsa M Ouzzani M. Rayyan: a systematic reviews web app for exploring and filtering searches for eligible studies for Cochrane Reviews. In: Evidence-Informed Publich Health: Opportunities and Challenges. Abstracts of the 22nd Cochrane Colloquium. John Wiley & Sons (2014).

[15] Kmet LM Lee RC Cook LS Alberta Heritage Foundation for Medical Research. Standard Quality Assessment Criteria for Evaluating Primary Research Papers From a Variety Of Fields. Edmonton, AB: Alberta Heritage Foundation for Medical Research (2004).

[16] Ancy J Gangadhar BN Janakiramaiah N. ‘Normal' P300 amplitude predicts rapid response to ECT in melancholia. J Affect Disord. (1996) 41:211–5. 10.1016/S0165-0327(96)00090-0 8988453

[17] Nurminen M Valkonen-Korhonen M Mervaala E Pääkkönen A Partanen J Viinamäki H. Enhanced attention-dependent auditory processing by electroconvulsive therapy in psychotic depression. J ECT. (2005) 21:19–24. 10.1097/01.yct.0000158015.88677.bc 15791173

[18] Miyauchi E Ide M Tachikawa H Nemoto K Arai T Kawasaki M. A novel approach for assessing neuromodulation using phase-locked information measured with TMS-EEG. Sci Rep. (2019) 9:428. 10.1038/s41598-018-36317-z 30674902 PMC6344580

[19] Grunhaus L Shipley JE Eiser A Pande AC Tandon R Remen A. Polysomnographic studies in patients referred for ECT: Pre-ECT studies. Convuls Ther. (1996) 12:224–31. 9034697

[20] Spitzer RL. Research diagnostic criteria: rationale and reliability. Arch Gen Psychiatry. (1978) 35:773 10.1001/archpsyc.1978.01770300115013 655775

[21] Göder R Hinrichsen I Seeck-Hirschner M Pfeiffer R Weinhold SL Baie PC. Sleep at baseline and after electroconvulsive therapy in patients with major depression. Psychiatry Res. (2016) 246:683–7. 10.1016/j.psychres.2016.10.064 27825788

[22] Hein M Lanquart J-P Loas G Hubain P Linkowski P. Alterations of neural network organisation during rapid eye movement sleep and slow-wave sleep in major depression: implications for diagnosis, classification, and treatment. Psychiatry Res Neuroimaging. (2019) 291:71–8. 10.1016/j.pscychresns.2019.08.003 31416044

[23] Drake ME Shy KE. Predictive value of electroencephalography for electroconvulsive therapy. Clin Electroencephalogr. (1989) 20:55–7. 10.1177/155005948902000112 2924428

[24] Roemer RA Shagass C Dubin W Jaffe R Katz R. Relationship between pretreatment electroencephalographic coherence measures and subsequent response to electroconvulsive therapy: a preliminary study. Neuropsychobiology. (1990) 24:121–4. 10.1159/000119473 2135066

[25] Scangos KW Weiner RD Coffey EC Krystal AD. An electrophysiological biomarker that may predict treatment response to ECT. J ECT. (2019) 35:95–102. 10.1097/YCT.0000000000000557 30531398 PMC6538443

[26] Stubbeman WF Leuchter AF Cook IA Shurman BD Morgan M Gunay I. Pretreatment neurophysiologic function and ECT response in depression. J ECT. (2004) 20:142–4. 10.1097/00124509-200409000-00004 15342997

[27] McCormick LM Yamada T Yeh M Brumm MC Thatcher RW. Antipsychotic effect of electroconvulsive therapy is related to normalization of subgenual cingulate theta activity in psychotic depression. J Psychiatr Res. (2009) 43:553–60. 10.1016/j.jpsychires.2008.08.004 18851858

[28] Kirsten A Seifritz E Olbrich S. Electroencephalogram source connectivity in the prediction of electroconvulsive therapy outcome in major depressive disorder. Clin EEG Neurosci. (2020) 51:10–8. 10.1177/1550059419888338 31752533

[29] Aiyer R Novakovic V Barkin RL. A systematic review on the impact of psychotropic drugs on electroencephalogram waveforms in psychiatry. Postgrad Med. (2016) 128:656–64. 10.1080/00325481.2016.1218261 27467441

[30] Bahji A Hawken ER Sepehry AA Cabrera CA Vazquez G. ECT: beyond unipolar major depression: systematic review and meta-analysis of electroconvulsive therapy in bipolar depression. Acta Psychiatr Scand. (2018) 139:214–26. 10.1111/acps.12994 30506992

[31] Picton T. The P300 wave of the human event-related potential. J Clin Neurophysiol. (1992) 9:456–79. 10.1097/00004691-199210000-00002 1464675

[32] Shahaf G. Neuropsychiatric disorders as erratic attention regulation – lessons from electrophysiology. Psychiatr Q. (2019) 90:793–801. 10.1007/s11126-019-09664-x 31410724

[33] Shahaf G. A possible common neurophysiologic basis for MDD, bipolar disorder, and schizophrenia: lessons from electrophysiology. Front Psychiatry. (2016) 7:94. 10.3389/fpsyt.2016.00094 27313546 PMC4887471

[34] Karaaslan F Gonul AS Oguz A Erdinc E Esel E. P300 changes in major depressive disorders with and without psychotic features. J Affect Disord. (2003) 73:283–287. 10.1016/S0165-0327(01)00477-3 12547298

[35] Jaworska N De Somma E Blondeau C Tessier P Norris S Fusee W. Auditory P3 in antidepressant pharmacotherapy treatment responders, non-responders and controls. Eur Neuropsychopharmacol. (2013) 23:1561–9. 10.1016/j.euroneuro.2013.03.003 23664712 PMC3744638

[36] Palagini L Baglioni C Ciapparelli A Gemignani A Riemann D. REM sleep dysregulation in depression: state of the art. Sleep Med Rev. (2013) 17:377–90. 10.1016/j.smrv.2012.11.001 23391633

[37] Voegeli G Cléry-Melin ML Ramoz N Gorwood P. Progress in elucidating biomarkers of antidepressant pharmacological treatment response: a systematic review and meta-analysis of the last 15 years. Drugs. (2017) 77:1967–86. 10.1007/s40265-017-0819-9 29094313

[38] Pillai V Kalmbach DA Ciesla JA. A Meta-analysis of electroencephalographic sleep in depression: evidence for genetic biomarkers. Biol Psychiatry. (2011) 70:912–9. 10.1016/j.biopsych.2011.07.016 21937023

[39] Shaw JC. An introduction to the coherence function and its use in EEG signal analysis. J Med Eng Technol. (1981) 5:279–88. 10.3109/03091908109009362 7328624

[40] Knyazev GG. EEG delta oscillations as a correlate of basic homeostatic and motivational processes. Neurosci Biobehav Rev. (2012) 36:677–95. 10.1016/j.neubiorev.2011.10.002 22020231

[41] Iosifescu DV. Electroencephalography-derived biomarkers of antidepressant response. Harv Rev Psychiatry. (2011) 19:144–54. 10.3109/10673229.2011.586549 21631160

[42] Leuchter AF Cook IA Lufkin RB Dunkin J Newton TF Cummings JL. Cordance: a new method for assessment of cerebral perfusion and metabolism using quantitative electroencephalography. NeuroImage. (1994) 1:208–19. 10.1006/nimg.1994.1006 9343572

[43] Ward MJ Karim HT Jessen ZF Ghuman AS Richardson RM Reynolds CF. Association between increased theta cordance and early response to ECT in late-life depression. Int J Geriatr Psychiatry. (2020) 35:147–52. 10.1002/gps.5220 31617234 PMC7047608

[44] Pizzagalli D Pascual-Marqui RD Nitschke JB Oakes TR Larson CL Abercrombie HC. Anterior cingulate activity as a predictor of degree of treatment response in major depression: evidence from brain electrical tomography analysis. Am J Psychiatry. (2001) 158:405–15. 10.1176/appi.ajp.158.3.405 11229981

